# Landscape of N^6^-methyladenosine (m^6^A) methylation in porcine cells identifies candidate genes associated with porcine epidemic diarrhea virus infection

**DOI:** 10.3389/fmicb.2026.1828985

**Published:** 2026-05-18

**Authors:** Xia Dong, Yue Zhang, Ying Wang, Cheng Wang, Ao Zhou

**Affiliations:** 1 Laboratory of Genetic Breeding, Reproduction and Precision Livestock Farming, School of Animal Science and Nutritional Engineering, Wuhan Polytechnic University, Wuhan, China; 2 Hubei Provincial Center of Technology Innovation for Domestic Animal Breeding, Wuhan, China; 3 Xiangxi Tujia and Miao Autonomous Prefecture Animal Husbandry and Aquatic Products Affairs Center, Jishou, Hunan, China

**Keywords:** immune response, m^6^A methylation, MeRIP-seq, PEDV, RNA-seq

## Abstract

Porcine epidemic diarrhea virus (PEDV) infection leads to serious intestinal disease in piglets, often leading to high mortality rates and substantial economic losses. Understanding host–PEDV interactions is crucial for PEDV therapeutic strategies. N^6^-methyladenosine (m^6^A) methylation has been proven to play an important role in host antiviral immunity. However, transcriptome-wide profiling patterns and the biological functions of host m^6^A methylation in response to PEDV infection remain incompletely understood. This study first observed significant upregulation of m^6^A regulators (METTL3, FTO, WTAP, YTHDC1, and YTHDF2) in PEDV infection. Following transcriptome-wide m^6^A methylation and gene expression profiling, this study identified 803 differentially methylated peaks with 674 differentially expressed m^6^A-methylated genes and 345 differentially expressed genes (DEGs) after PEDV infection. The Gene Ontology (GO) and Kyoto Encyclopedia of Genes and Genomes (KEGG) analyses indicated that these differentially methylated genes were enriched mainly in lysine degradation, histidine metabolism, and the ubiquitin-mediated proteolysis pathway, whereas these DEGs were enriched in negative regulation of viral genome replication, viral protein interaction with cytokines and cytokine receptors, nucleotide-binding oligomerization domain-like (NOD-like) receptor signaling, and immune response-related signaling pathways. Furthermore, the joint analysis of RNA sequencing (RNA-seq) and methylated RNA immunoprecipitation sequencing (MeRIP-seq) identified 16 differentially expressed genes with m^6^A methylation (*AMDHD1, CPM, DCTPP1, GIMAP1, GVIN1, HERC6, LOC100522040, LOC106510546, NFAT5, PIM3, PPARGC1B, RASSF2, SAPCD2, URB2, XRRA1,* and *ZC3HAV1L*), which were associated with immune response and metabolism. Taken together, the study results map the dynamic landscape of host m^6^A methylation and demonstrate the functional enrichment of m^6^A methylated genes during PEDV infection, thereby providing a theoretical framework for future research on the role of m^6^A methylation in resistance to PEDV infection.

## Introduction

Porcine epidemic diarrhea virus (PEDV) is a highly contagious intestinal coronavirus that causes acute watery diarrhea, vomiting, dehydration, and high morbidity and mortality in neonatal piglets, leading to substantial economic losses in the global pig industry (Jung et al., 2020; Park, 2024). As a single-stranded RNA-enveloped coronavirus, PEDV exhibits high mutation rates with varying virulence to facilitate evasion and adaptation for replication (Zhang et al., 2023). Strict biosecurity measures, reasonable immunization strategies, and accurate pathogen detection are useful for preventing and controlling PEDV infection; however, the pathogenesis of PEDV and host–PEDV interactions remain incompletely understood, limiting the development of effective treatments against PEDV infection.

Epigenetic modifications have been shown to regulate viral replication and disease progression (Rabaan et al., 2023). N^6^-methyladenosine (m^6^A) methylation is the most abundant and reversible internal RNA modification. m^6^A methylation is dynamically regulated by methyltransferase writers, readers, and erasers (Jiang et al., 2021), and is predominantly localized near stop codons and 3′-UTRs within conserved motifs such as RRACH (Huang et al., 2021). Previous studies have shown that m^6^A methylation plays significant regulatory roles in physiological and pathological processes, including diseases occurrence, viral infection, inflammation, and antiviral immunity, primarily through its effects on RNA stability and degradation, alternative splicing, translation efficiency, and nuclear export (Feng et al., 2021; Wang et al., 2022). m^6^A modifications affect the export of hepatitis B virus (HBV) RNAs to regulate the HBV life cycle (Kim et al., 2021). Enterovirus 71 (EV71) RNA-dependent RNA polymerase 3D interacts with the m^6^A writer METTL3 to increase the SUMOylation and ubiquitination of 3D polymerase, boosting viral replication (Hao et al., 2019). Influenza A virus infection induces the expression of the m^6^A reader protein YTHDC1, which binds to the NS splicing site with the m^6^A motif GGA(530)C to inhibit NS1 splicing, subsequently promoting IAV replication and pathogenicity (Zhu et al., 2023). Classical swine fever virus (CSFV) non-structural protein 5B interacts with the E3 ubiquitin ligase of METTL14 to prevent METTL14 degradation, leading to TLR4 mRNA decay facilitated by YTHDF2 with a m^6^A-dependent pattern (Chen et al., 2024). Foot-and-mouth disease virus (FMDV) structural protein VP1 promotes autophagy, inducing degradation of the m^6^A reader YTHDF2, which increases GTPBP4 expression and suppresses type I interferon production (Liu et al., 2024). Additionally, in response to viral infection, host cells may impair the enzymatic activity of the RNA m^6^A demethylase ALKBH5, resulting in the reduction of OGDH mRNA stability and protein expression for decreasing the production of the metabolite itaconate required for viral replication (Liu et al., 2019). Additional studies have highlighted that an E3 small ubiquitin-like modifier (SUMO) ligase PIAS1 mediates SUMOylation of the m^6^A readers YTHDFs (YTHDF1, 2, and 3), which increases the binding to EBV transcripts, leading to decreased viral mRNA stability and restricted EBV replication (Sugiokto et al., 2024). Host m^6^A-IRF7-IFN antiviral signaling cascade restricts rotavirus infection *in vivo* (Wang et al., 2022). Heterogeneous nuclear ribonucleoprotein A2B1 (hnRNPA2B1) regulates m^6^A methylation of *CGAS, IFI16,* and *STING* genes and their nucleocytoplasmic trafficking to increase IFN-α/β production and activate antiviral signaling (Wang et al., 2019). These findings suggest that m^6^A methylation is closely associated with host antiviral immunity.

This study performed transcriptome-wide m^6^A-methylation sequencing to investigate the role of m^6^A methylation in porcine cells following PEDV infection. These results revealed significant alterations in the m^6^A methylation landscape between PEDV-infected and control cells. Notably, genes exhibiting differential m^6^A methylation were predominantly enriched in glycosaminoglycan biosynthesis and lysine degradation pathways. Collectively, this study provides novel insights into the regulatory role of m^6^A methylation in host gene expression during PEDV infection.

## Materials and methods

### Cell and virus infection

The porcine kidney proximal tubular epithelial cell line (LLC-PK1) (Procell, Wuhan, China), free from mycoplasma, was maintained in Dulbecco’s modified Eagle’s medium (DMEM) supplemented with 10% fetal bovine serum (FBS, CELLBOX, Changsha Cell-Box Biotechnology Co., Ltd., Changsha, Hunan, China), and 1% penicillin–streptomycin solution (Gibco, Thermo Fisher Scientific, Waltham, MA, USA) under a humidified 5% carbon dioxide atmosphere at 37 °C. LLC-PK1 cells were selected for this study because they are permissive for PEDV infection and have been widely used as an *in vitro* model for PEDV research. Additionally, this cell line is easier to culture and manipulate, making it suitable for initial mechanistic studies. PEDV YN strain (GenBank accession No. KT021228) stored in the laboratory was propagated using Vero cells (renal cells from African green monkey, Procell, Wuhan, China) in DMEM supplemented with 5 μg/mL trypsin and 1% penicillin–streptomycin solution. In addition, LLC-PK1 cells were cultivated in 6-well culture plates at a density of approximately 5 × 10^5^ cells per well until a 100% confluence monolayer was reached. Subsequently, the cells were washed with PBS and were infected with PEDV (MOI = 0.01). To investigate the early host m^6^A methylation response to PEDV infection, western blot analysis was performed at 0, 9, and 12 h post-infection (hpi). For transcriptome-wide m^6^A methylation and gene expression profiling, the cells were collected at 24 hpi to capture broad transcriptional changes following PEDV infection. All experiments using the virus were performed in a biosafety level 2 (BSL2) laboratory.

### Western blotting

Porcine cells infected with PEDV were collected at 9 and 12 hpi and extracted using a radioimmunoprecipitation assay (RIPA) buffer supplemented with phenylmethanesulfonyl fluoride (PMSF) and a phosphatase inhibitor. After quantification the samples using a BCA Protein Reagent Assay Kit, they were denatured, loaded on to a 10% SDS-PAGE gel, subjected to electrophoresis, and then transferred to the polyvinylidene fluoride membrane (PVDF) after blocking with 5% BSA for 60 min at room temperature. The blocked membranes were incubated overnight with primary antibodies (METTL3, FTO, WTAP, YTHDC1, YTHDF2, and YTHDF3 antibodies, Proteintech, Wuhan, China) at 4 °C. The membranes were subsequently incubated with anti-rabbit IgG HRP-linked antibody (dilution 1:10,000, Proteintech, Wuhan, China) for 2 h at room temperature and were visualized using an infrared imaging system. Glyceraldehyde-3-phosphate dehydrogenase (GAPDH) was used as an internal loading control for normalization. Each experiment was performed twice with similar results, and representative blots are shown. ImageJ software was used to quantify the results obtained from western blotting.

### MeRIP-seq and RNA-seq libraries construction

After 24 hpi with PEDV, total RNA from each group with three biological replicates was extracted from LLC-PK1 cells using the TRIzol reagent following the manufacturer’s instructions and then quantified using NanoDrop based on the OD260/280 ratio. In addition, RNA integrity was assessed with denaturing agarose gel electrophoresis and the Agilent 2100 bioanalyzer using the RNA integrity number (RIN) value (Agilent Technologies, Santa Clara, CA, United States). Oligo d(T) magnetic beads were used to purify mRNA. The enriched mRNA was then randomly fragmented into 100–150 nucleotide-long pieces using a magnesium RNA fragmentation module. The fragmented poly(A) RNAs were immunoprecipitated using the m^6^A-specific antibody (Synaptic Systems, 202203) to construct MeRIP-seq libraries, while the stranded RNA-seq libraries were constructed using the stranded mRNA Library Prep Kit for Illumina^®^ (Catalog No. DR08402) following the manufacturer’s instructions. The MGISEQ-T7 platform was used to carry out double-ended sequencing following the standard operating procedures in the 2 × 150 bp paired-end sequencing (PE150) model. Trimmomatic (version 0.36) software (Bolger et al., 2014) was used to filter low-quality reads, including those containing adaptor contamination and undetermined bases.

### Data processing and bioinformatics analysis

For MeRIP-seq, the clean reads of all samples were mapped to the porcine reference genome (Sscrofa11.1) using STAR software (version 2.5.3a) (Dobin et al., 2013) with default parameters before peak calling was performed using exomePeak (version 3.8) with *p* < 0.05 across the whole genome. Peaks with >50% overlap were retained for subsequent analysis. The annotation of m^6^A peaks and peak distribution analysis were performed using BEDTools (Version 2.25.0) (Quinlan and Hall, 2010) and deepTools (version 2.4.1), respectively. Differential m^6^A peaks analysis was conducted using the exomePeak (Version 3.8) based on the following criteria: adjusted *p*-value <0.05 and |log2(fold change)| ≥1. The peaks were visualized using Integrated Genome Viewer (IGV), and sequence motifs enriched in m^6^A peak regions were identified using HOMER (version 4.10) (Heinz et al., 2010).

For RNA-seq, STAR software (version 2.5.3a) was used to map the clean reads into the porcine reference genome (Sscrofa11.1) using default parameters. DESeq (Love et al., 2014) was used to identify differentially expressed genes with the cut-offs of |log2(fold change) >1| and *p*-value of <0.05.

### qRT-PCR

Total RNA was extracted from three independent biological replicates using TRIzol reagent according to the manufacturer’s instructions, and the quality of RNA was detected using NanoDrop based on the OD260/280 ratio and denaturing agarose gel electrophoresis. The RNA samples were then synthesized into first-strand cDNA using the TaKaRa reverse transcription kit for quantitative reverse transcription polymerase chain reaction (qRT-PCR) reactions using gene primers (Supplementary Table S1). Relative gene expression levels were obtained using the 2^−ΔΔCT^ method, with β-actin serving as the internal reference.

### Statistical analysis

**Figure 1:**
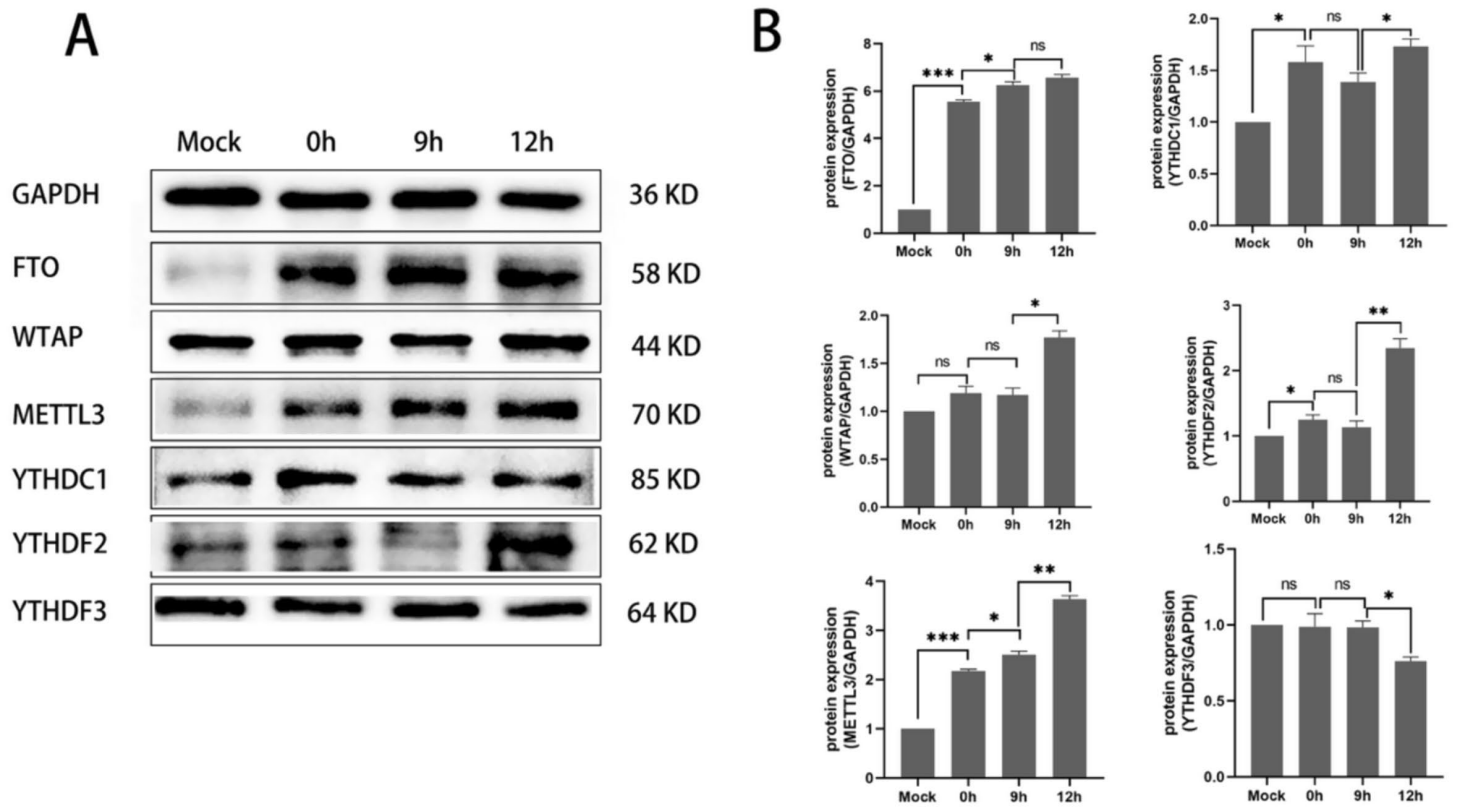
m^6^A methylation-related enzymes expression analysis. **(A)** Expression of methylation-related enzymes was detected by Western blotting using the indicated antibodies. GAPDH was used as a loading control. **(B)** Gray scale quantitative analysis. Three independent experiments were performed, and data are shown as the mean ± SD. The statistical significance was determined using the two-tailed Student’s *t*-test. ^*^p < 0.05, ^**^p < 0.01, and ^***^p < 0.001.

For comparisons between two groups, data were analyzed using an unpaired Student’s *t*-test. For comparisons among multiple groups (Mock, 0 h, 9 h, and 12 h; see Figure 1), one-way analysis of variance (ANOVA), followed by Tukey’s multiple comparison test, was performed. All statistical analyses were conducted using GraphPad Prism (version 8.0). Each group consisted of three biological replicates, and data are presented as mean ± standard deviation (SD). A *p*-value of <0.05 was considered statistically significant.

## Results

### m^6^A methylation-related enzyme expression

To investigate the effect of m^6^A modification on PEDV-infected porcine cells, the protein expression levels of m^6^A methylation-related enzymes were detected by western blotting. The results showed that the expression of METTL3, FTO, WTAP, YTHDC1, and YTHDF2 was significantly increased at the protein level during PEDV infection, whereas PEDV infection led to a decrease in the expression of YTHDF3 (Figures 1A,B). These results suggest that m^6^A methylation modifications may be involved in PEDV infection.

### Global dynamic change patterns of m^6^A methylation in porcine cells after PEDV infection

To analyze the biological processes of m^6^A methylation in porcine cells infected with PEDV, this study performed a transcriptome-wide MeRIP-seq analysis and found that an average of 10.3 Gb of sequencing data in the MeRIP-seq samples and 7.8 Gb of sequencing data in the input samples were generated (Table 1). After statistical analysis and quality control, the effective percentages of clean reads (Q30) were greater than 88% in the immunoprecipitation (IP) libraries and 84% in the input libraries, indicating that the collected data were clean, and the average number of clean data was 50 million in the IP libraries and 40 million in the input libraries. Moreover, more than 90% of the reads were mapped to the porcine reference genome (*Sus scrofa*) (Table 2), and over 60% of the mapped reads were aligned to exon regions in the control and PEDV infection groups (Additional file 1).

**Table 1:** Detail information of MeRIP-seq.

Sample	Raw reads	Raw bases (G)	Raw Q20 (%)	Raw Q30 (%)	Raw GC (%)	Clean reads	Clean bases (G)	Clean Q20 (%)	Clean Q30 (%)	Clean GC (%)	Effective rate (%)
Ctr_1_IP	68,093,074	10.21	99.16	96.85	51.95	63,139,822	7.96	99.62	98.09	51.72	92.73
Ctr_1_Input	66,273,620	9.94	99.03	96.35	51.23	57,841,206	7.67	99.54	97.68	51.08	87.28
Ctr_2_IP	76,272,814	11.44	99.18	96.9	51.77	70,587,176	8.88	99.62	98.08	51.67	92.55
Ctr_2_Input	52,513,740	7.88	99.03	96.39	51.05	46,370,210	6.26	99.55	97.74	50.75	88.3
Ctr_3_IP	69,440,934	10.42	99.08	96.57	52.27	63,364,530	8.07	99.59	97.93	51.95	91.25
Ctr_3_Input	56,029,268	8.4	98.97	96.12	51.03	48,506,008	6.55	99.51	97.53	50.7	86.57
PEDV_1_IP	68,860,974	10.33	99.18	96.96	51.57	61,467,660	7.79	99.65	98.21	51.56	89.26
PEDV_1_Input	45,197,666	6.78	98.92	96.12	51.12	37,882,234	5.02	99.57	97.82	50.82	83.81
PEDV_2_IP	68,252,858	10.24	99.15	96.85	51.94	59,063,396	7.46	99.63	98.14	51.65	86.54
PEDV_2_Input	46,094,698	6.91	98.91	96.06	51.04	40,806,798	5.55	99.56	97.77	50.9	88.53
PEDV_3_IP	66,585,974	9.99	99.21	97.06	51.56	55,736,292	7.01	99.66	98.27	51.56	83.71
PEDV_3_Input	41,940,256	6.29	98.97	96.31	51.3	35,988,628	4.77	99.6	97.98	50.93	85.81

**Table 2:** Statistical analysis of reads mapped in the porcine reference genome for MeRIP-seq.

Sample	Total_reads	Total_mapped (%)	Non_unique (%)	Unique (%)	Un_mapped_reads (%)	Read1 (%)	Read2 (%)	Reads_map_plus (%)	Reads_map_minus (%)	Non_splice_reads (%)	Splice_reads (%)
Ctr_1_IP	57,985,018	53,389,229 (92.07)	1,522,307 (2.85)	51,866,922 (97.15)	4,595,789 (7.93)	25,933,467 (50.0)	25,933,455 (50.0)	25,933,458 (50.0)	25,933,464 (50.0)	35,494,428 (68.43)	16,372,494 (31.57)
Ctr_1_Input	53,510,046	48,608,917 (90.84)	1,239,037 (2.55)	47,369,880 (97.45)	4,901,129 (9.16)	23,684,967 (50.0)	23,684,913 (50.0)	23,684,936 (50.0)	23,684,944 (50.0)	26,355,962 (55.64)	21,013,918 (44.36)
Ctr_2_IP	64,929,752	59,774,361 (92.06)	1,717,671 (2.87)	58,056,690 (97.13)	5,155,391 (7.94)	29,028,350 (50.0)	29,028,340 (50.0)	29,028,343 (50.0)	29,028,347 (50.0)	39,276,559 (67.65)	18,780,131 (32.35)
Ctr_2_Input	42,970,174	38,878,385 (90.48)	959,458 (2.47)	37,918,927 (97.53)	4,091,789 (9.52)	18,959,495 (50.0)	18,959,432 (50.0)	18,959,460 (50.0)	18,959,467 (50.0)	21,078,438 (55.59)	16,840,489 (44.41)
Ctr_3_IP	58,263,630	53,559,405 (91.93)	1,546,903 (2.89)	52,012,502 (97.11)	4,704,225 (8.07)	26,006,265 (50.0)	26,006,237 (50.0)	26,006,251 (50.0)	26,006,251 (50.0)	35,718,102 (68.67)	16,294,400 (31.33)
Ctr_3_Input	44,829,090	40,581,609 (90.53)	1,002,126 (2.47)	39,579,483 (97.53)	4,247,481 (9.47)	19,789,771 (50.0)	19,789,712 (50.0)	19,789,744 (50.0)	19,789,739 (50.0)	21,976,483 (55.52)	17,603,000 (44.48)
PEDV_1_IP	55,993,968	50,728,737 (90.6)	1,488,527 (2.93)	49,240,210 (97.07)	5,265,231 (9.4)	24,620,108 (50.0)	24,620,102 (50.0)	24,620,102 (50.0)	24,620,108 (50.0)	34,187,656 (69.43)	15,052,554 (30.57)
PEDV_1_Input	34,096,206	30,818,483 (90.39)	874,496 (2.84)	29,943,987 (97.16)	3,277,723 (9.61)	14,971,994 (50.0)	14,971,993 (50.0)	14,971,992 (50.0)	14,971,995 (50.0)	16,904,840 (56.45)	13,039,147 (43.55)
PEDV_2_IP	54,057,152	49,030,452 (90.7)	1,465,842 (2.99)	47,564,610 (97.01)	5,026,700 (9.3)	23,782,308 (50.0)	23,782,302 (50.0)	23,782,305 (50.0)	23,782,305 (50.0)	32,864,910 (69.1)	14,699,700 (30.9)
PEDV_2_Input	36,822,718	33,413,360 (90.74)	813,980 (2.44)	32,599,380 (97.56)	3,409,358 (9.26)	16,299,693 (50.0)	16,299,687 (50.0)	16,299,691 (50.0)	16,299,689 (50.0)	17,676,892 (54.22)	14,922,488 (45.78)
PEDV_3_IP	51,218,680	46,528,926 (90.84)	1,450,031 (3.12)	45,078,895 (96.88)	4,689,754 (9.16)	22,539,449 (50.0)	22,539,446 (50.0)	22,539,447 (50.0)	22,539,448 (50.0)	31,272,717 (69.37)	13,806,178 (30.63)
PEDV_3_Input	32,486,910	29,438,218 (90.62)	815,990 (2.77)	28,622,228 (97.23)	3,048,692 (9.38)	14,311,118 (50.0)	14,311,110 (50.0)	14,311,116 (50.0)	14,311,112 (50.0)	15,999,471 (55.9)	12,622,757 (44.1)

**Figure 2:**
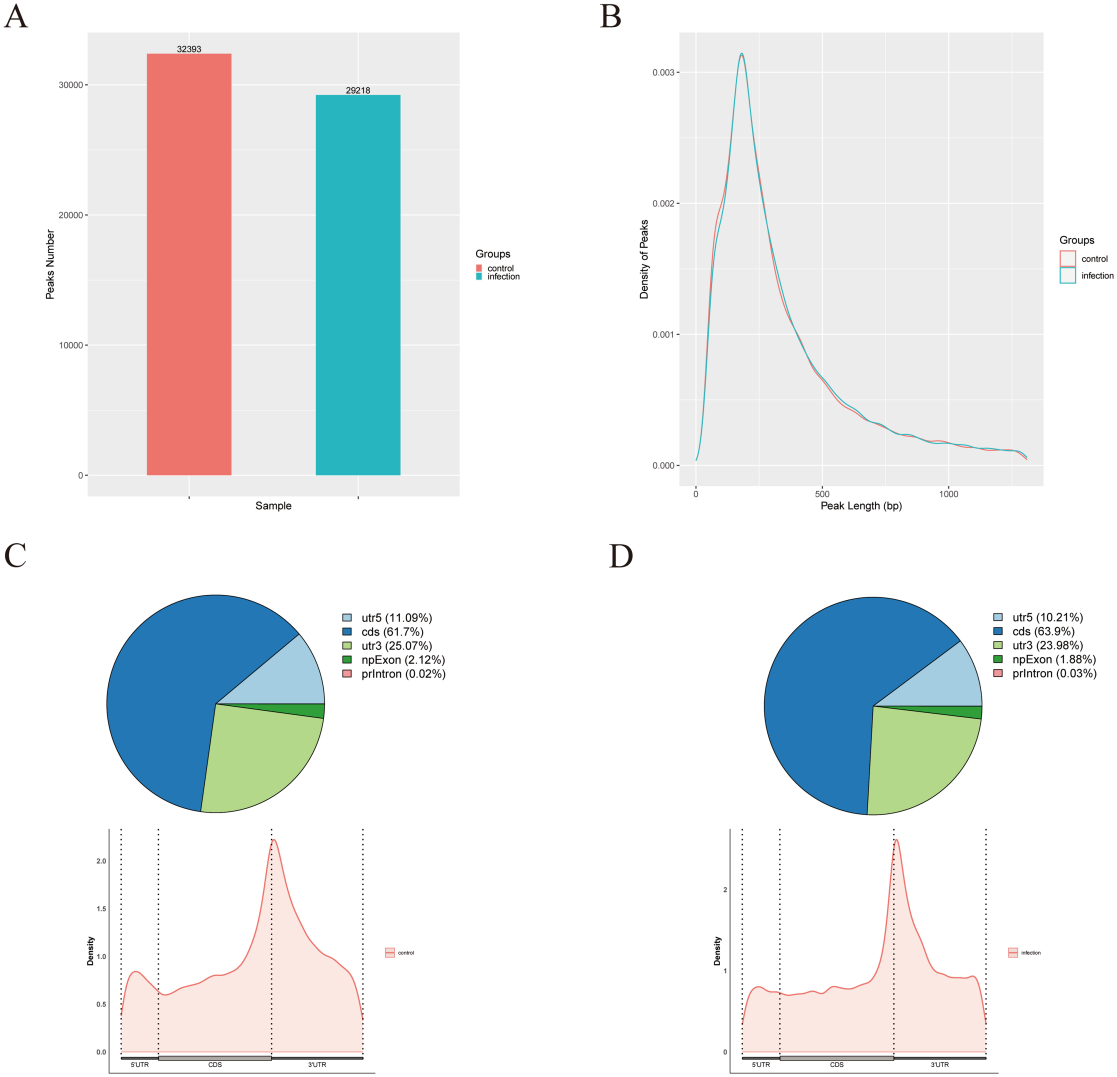
Overview of m^6^A peaks distribution landscape. **(A)** The average number of m^6^A peaks in the control group and the PEDV-infected group. **(B)** The density of m^6^A peaks in the control group and the PEDV-infected group. **(C)** m^6^A peak distributions in different gene functional elements (5′ UTR, 3′ UTR, CDS, and intron) in the control group. **(D)** m^6^A peak distributions in different gene functional elements (5′ UTR, 3′ UTR, CDS, and intron) in the PEDV-infected group.

**Figure 3:**
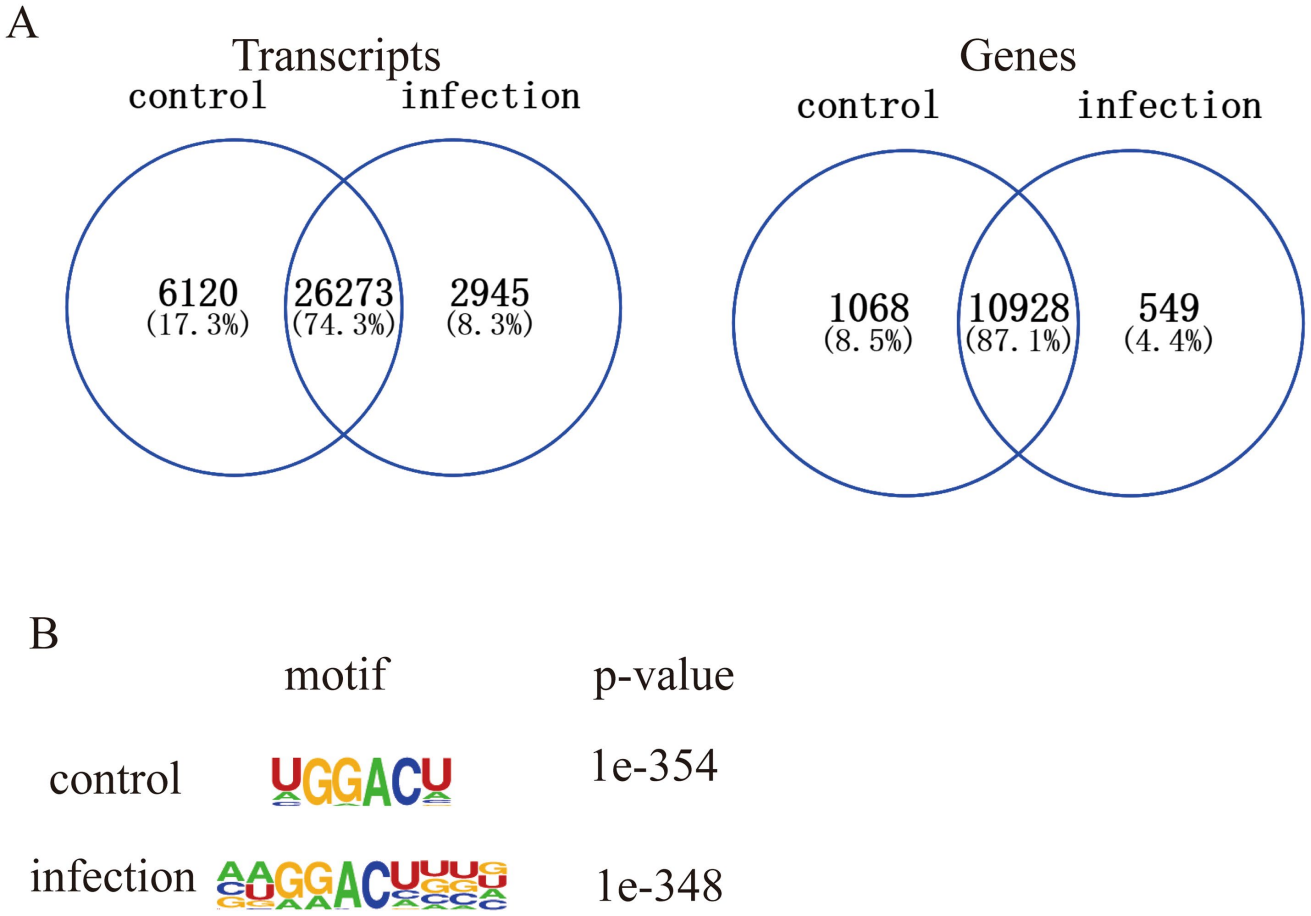
m^6^A peak distribution analysis between the control group and the PEDV-infected group. **(A)** The overlapped transcripts and genes of m^6^A peaks between control and PEDV-infected groups. **(B)** The most conserved sequence motif of the differential m^6^A peak region in control and PEDV-infected groups.

Next, this study analyzed the topological distribution of m^6^A peaks at the genome level. The results showed that a total of 32,393 and 29,218 m^6^A peaks with high confidence were distributed across 11,996 and 11,477 genes, respectively (Figure 2A). In addition, this study found that the average length of the peaks between control and PEDV-infected cells showed no difference (Figure 2B). Moreover, 60% of m^6^A peaks were enriched in the coding sequences (CDS) region, but m^6^A peaks had the highest density in the stop codon region (Figures 2C,D). Furthermore, the Venn diagram showed that the distribution patterns of m^6^A peak numbers with genes were highly similar, with 26,273 common m^6^A peaks related to 10,928 genes in both groups (Figure 3A). Concomitantly, the HOMER analysis showed the top consensus motif identified in both groups was the “GGACUU” motif, which had the highest scores (Figure 3B). In summary, these results indicated m^6^A distribution and motifs in porcine cells are conserved and that m^6^A methylation patterns may differ between the control and PEDV-infection samples.

### Function enrichment analysis of genes presenting differential m^6^A peaks

**Figure 4:**
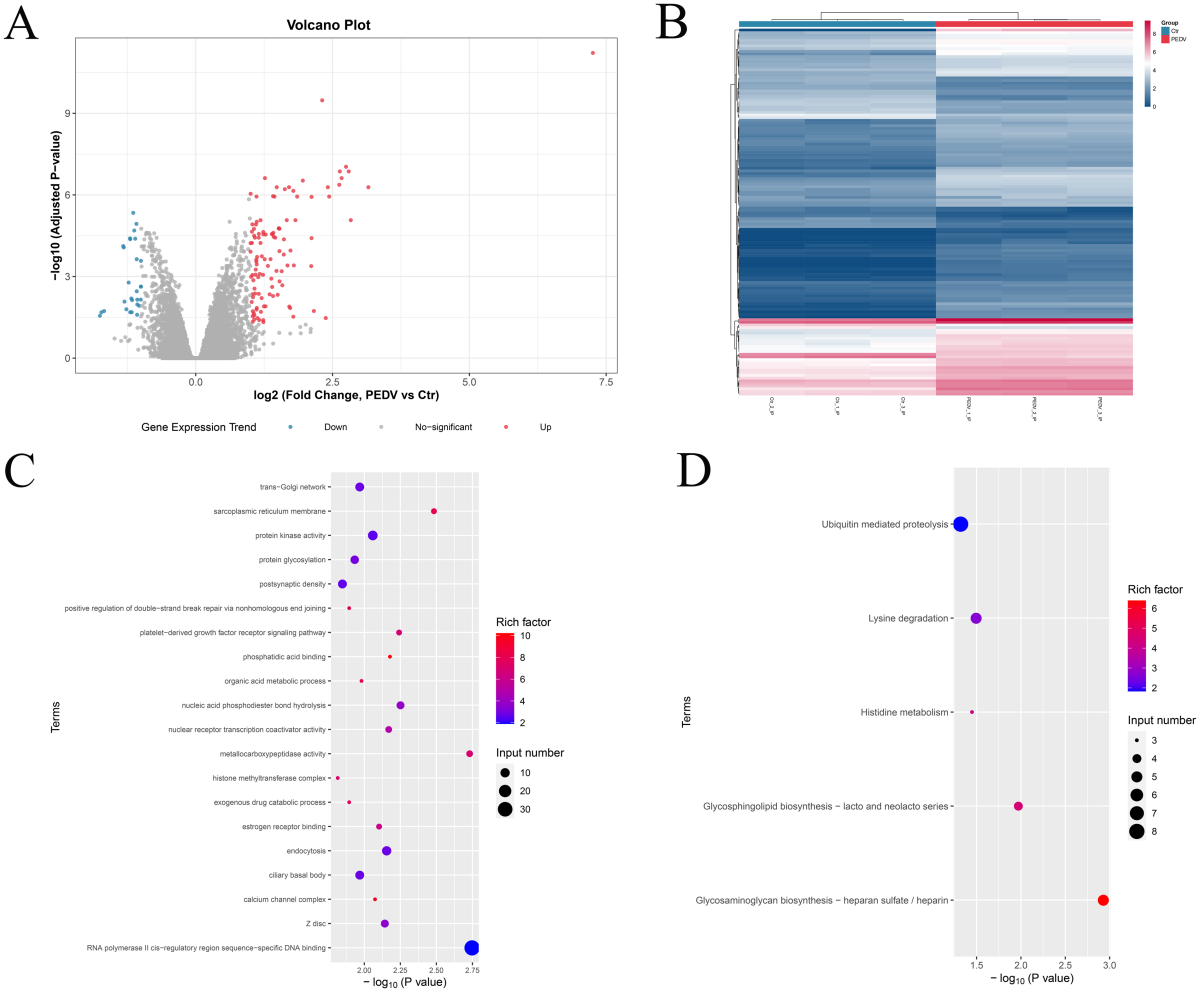
Differentially expressed m^6^A peaks and function analysis. **(A)** Volcano plot shows the differentially expressed m^6^A peaks in the PEDV group versus the control group. **(B)** Heatmap shows the patterns of the differentially expressed m^6^A methylation genes. **(C,D)** Gene Ontology and pathway enrichment analysis of differentially expressed methylated genes, respectively.

To reveal the role of m^6^A methylation during PEDV infection, the differential m^6^A peaks in the PEDV-infected and control groups were scanned. A total of 803 differentially methylated peaks were identified, including 327 hyper-methylated m^6^A peaks and 476 hypo-methylated m^6^A peaks (log2|fold change| >1.0, adjusted *p* < 0.05) (Figures 4A,B). Based on the annotated genes with differential peaks, these significantly differential m^6^A peaks were distributed in 674 DMGs (Additional file 2). Furthermore, GO enrichment analysis of these DMGs was performed to investigate the potential biological roles of DMGs in response to PEDV infection. The results demonstrated that DMGs were mainly involved in the trans-Golgi network, protein kinase activity, metabolic processes, and the platelet-derived growth factor receptor signaling pathway (Figure 4C). Meanwhile, KEGG pathway analysis revealed that DMGs were mainly enriched in glycosaminoglycan biosynthesis–heparan sulfate/heparin, glycosphingolipid biosynthesis–lacto and neolacto series, lysine degradation, histidine metabolism, and ubiquitin-mediated proteolysis (Figure 4D).

### Differentially expressed genes analysis by RNA-seq

**Figure 5:**
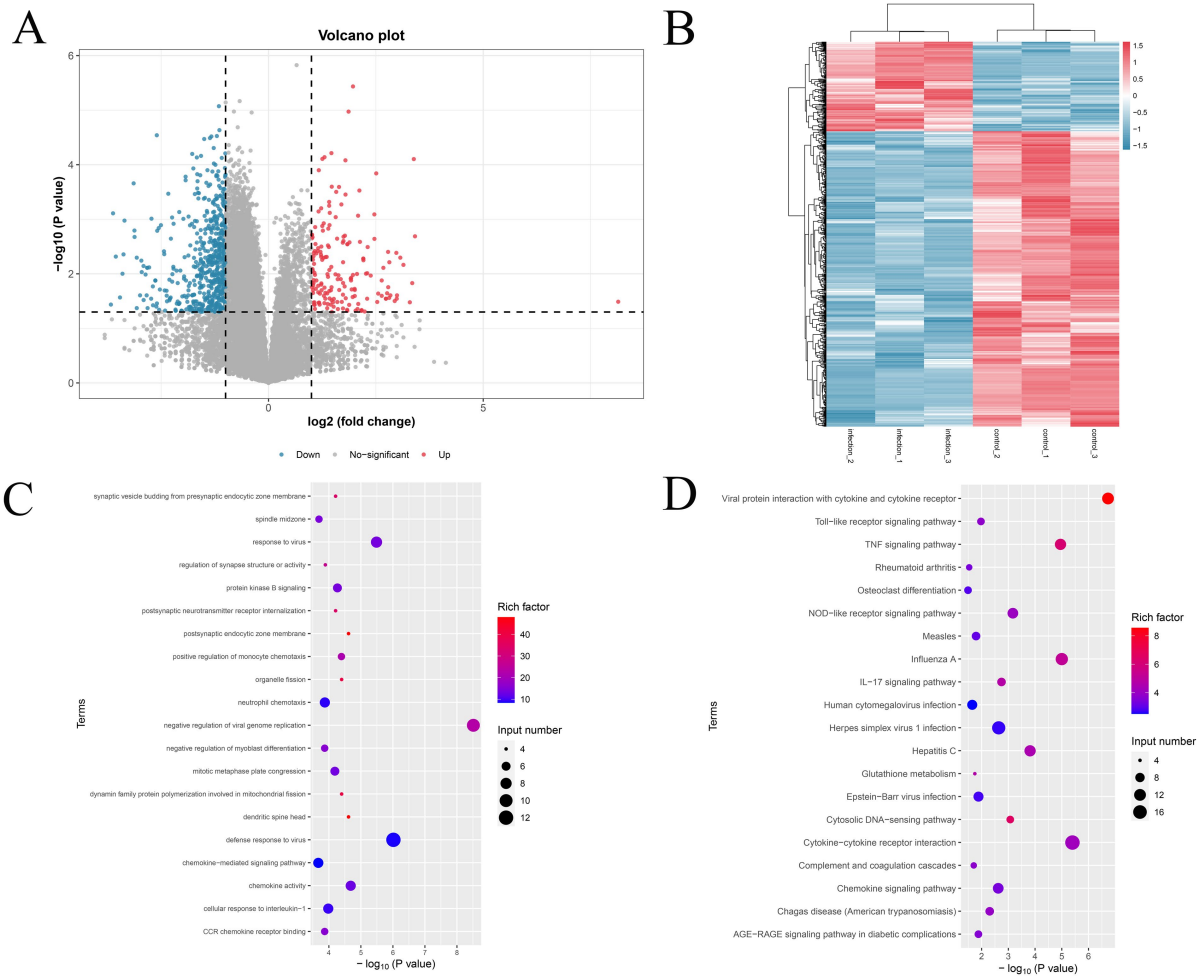
Differentially expressed genes and function analysis. **(A)** Volcano plot shows the differential gene expression between the control group and the PEDV-infected group. **(B)** Hierarchical cluster analysis of differentially expressed genes. **(C,D)** Gene Ontology and pathway enrichment analysis of differentially expressed genes, respectively.

To analyze the potential relationship between m^6^A modification and gene expression, differentially expressed genes (DEGs) were first identified using RNA-seq data, and the results are shown using the volcano plot and heatmap (Figures 5A,B). A total of 207 upregulated DEGs and 138 downregulated DEGs were identified during PEDV infection following the criteria: |log2(fold change)| >1 and adjusted *p*-value <0.05. The GO enrichment analysis showed that DEGs were mainly involved in biological processes, such as negative regulation of viral genome replication, defense response to virus, cellular response to interleukin-1, and other processes related to signaling transduction and immunity (Figure 5C). KEGG pathway analysis revealed that DEGs were mainly enriched in pathways associated with virus infection and host–virus interaction, including cytokine–cytokine receptor interaction, influenza A, TNF signaling, NOD-like receptor signaling, cytosolic DNA-sensing, and IL-17 signaling (Figure 5D).

### Association analysis of m^6^A methylation and gene expression after PEDV infection

**Figure 6:**
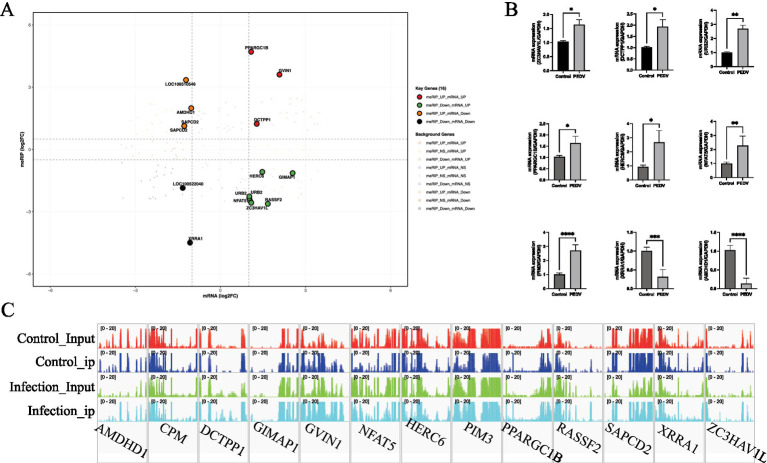
Integrated analysis of MeRIP-seq and RNA-seq data. **(A)** Venn diagram shows the overlap between differentially expressed methylated genes (DMGs) identified by MeRIP-seq and differentially expressed genes (DEGs) identified by RNA-seq. A total of 16 genes were identified with both differential methylation and differential expression. **(B)** qRT-PCR validation of the expression levels of selected genes with differential m^6^A methylation and differential expression. **(C)** Representative IGV tracks show m^6^A peaks of the candidate genes (illustrative; orthogonal validation such as MeRIP-qPCR would be required to confirm m^6^A enrichment).

To investigate the coregulatory relationship between transcript levels and m^6^A methylation in porcine cells in response to PEDV infection, this study performed an integrated analysis of MeRIP-seq data and RNA-seq data. The results showed that only 16 differentially methylated and expressed genes (*AMDHD1, CPM, DCTPP1, GIMAP1, GVIN1, HERC6, LOC100522040, LOC106510546, NFAT5, PIM3, PPARGC1B, RASSF2, SAPCD2, URB2, XRRA1*, and *ZC3HAV1L*) were identified (Figure 6A and Table 3). qRT-PCR confirmed the expression patterns of these genes, which were consistent with the transcriptomic data (Figure 6B). Representative Integrative Genomics Viewer (IGV) tracks showing m^6^A peaks of the candidate genes are provided as illustrative examples (Figure 6C); orthogonal validation (such as MeRIP-qPCR) will be required to confirm m^6^A enrichment.

**Table 3:** List of 16 genes with differential m^6^A modification identified by integrated MeRIP-seq and RNA-seq analysis.

Gene ID	Peak ID	MeRIP_diff_peaks_genes (logFC)	Adjust *p*-value	mRNA_diff_genes (logFC)	*p*-value
AMDHD1	Infection_peak_11373	1.987474181	0.0227363075972037	−1.028538477	1.47240659801666 × 10^−5^
CPM	Infection_peak_11052	1.111307008	0.0309780317429787	1.004613659	8.46620499012955 × 10^−10^
DCTPP1	Infection_peak_6040	1.233104984	0.0114787566163	1.28032275	3.3803963533699 × 10^−5^
GIMAP1	Control_peak_34151	−1.141305197	0.0155262815430849	2.545184133	8.39274425666378 × 10^−23^
GVIN1	Infection_peak_18681	3.605942974	0.0075201058806348	2.081189443	1.32260447278357 × 10^−8^
HERC6	Control_peak_20227	−1.082879572	0.036564642471023	1.477334832	1.02973671547731 × 10^−16^
LOC100522040	Control_peak_31738	−1.856162325	0.036070977	−1.327572739	2.60 × 10^−5^
LOC106510546	Infection_peak_12012	3.348528692	0.0145119724832567	−1.212220954	0.000510355302214148
NFAT5	Control_peak_14865	−2.472881928	0.0428589689205882	1.044746212	1.04699627289216 × 10^−18^
PIM3	Infection_peak_10687	1.639909753	0.0478883522872399	1.016809023	0.0147930737803513
PPARGC1B	Infection_peak_4339	4.704976805	0.0463049317395626	1.080057826	2.46942748411603 × 10^−11^
RASSF2	Control_peak_33228	−2.620985712	0.0133196274207615	1.670832144	1.97850317085457 × 10^−10^
SAPCD2	Infection_peak_32546	1.129317969	0.0036535168208805	−1.273481595	3.24108699668828 × 10^−13^
URB2	Infection_peak_27600	1.173901214	0.0446987571209284	1.017676139	1.37404165512868 × 10^−11^
XRRA1	Control_peak_21400	−4.495020014	0.040007547	−1.069986611	8.62 × 10^−7^
ZC3HAV1L	Control_peak_33803	−2.571458467	0.0432842240964784	1.086596408	0.00066733097168513

## Discussion

Infection by PEDV causes highly contagious intestinal disease and high mortality in piglets, leading to serious economic losses for the pig industry. Growing evidence has revealed that m^6^A methylation is involved in virus replication and infection (Kostyusheva et al., 2021; Wang et al., 2020). However, our understanding of how m^6^A methylation and its related pathways impacts the replication and spread of PEDV is limited. This study first measured the expression of m^6^A methylation regulators and found that the protein expression of m^6^A methylation regulators (METTL3, FTO, WTAP, YTHDC1, and YTHDF2) was significantly increased in PEDV infection, except for YTHDF3, which can accelerate the metabolism of m^6^A-modified mRNAs and can suppress interferon-dependent antiviral responses by accelerating the metabolism of m^6^A-modified mRNAs (Shi et al., 2017; Zhang et al., 2019). The findings suggest that the alteration in host m^6^A methylation is associated with PEDV infection.

To better understand the relationship between m^6^A methylation modification and gene expression in porcine cells in response to PEDV infection, this study determined the global landscape of m^6^A methylation on porcine cells’ transcriptome through MeRIP-seq and found that PEDV infection decreased the number of m^6^A peaks in cellular RNAs. The methylated m^6^A peaks were enriched in CDS and 3′-UTR regions, which was consistent with previous studies on the alterations in the m^6^A profile of mammalian cells upon virus infection (Lin et al., 2023). In addition, 674 differentially methylated genes were identified in the PEDV-induced group and mainly enriched in the trans-Golgi network, platelet-derived growth factor receptor signaling pathway, glycosaminoglycan biosynthesis, lysine degradation, histidine metabolism, and ubiquitin-mediated proteolysis. Viruses can utilize glycosaminoglycan for viral attachment into cells, and heparan sulfate (HS) is the most common ingredient in glycosaminoglycan and is found as an attachment factor of PEDV (Lee et al., 2006; Huan et al., 2015). In response, IGF2BP2 was shown to regulate aerobic glycolytic capacity, affecting the growth of cervical cancer caused by human papillomavirus infection (Hu et al., 2022). Moreover, m^6^A modification can regulate various aspects of RNA metabolism to affect antiviral innate immunity (Karandashov et al., 2024; Liu et al., 2019), suggesting that m^6^A methylation may mediate host cell abnormal metabolism in response to PEDV infection. In addition, this study found that PEDV infection altered porcine cell metabolism through regulating the expression of metabolism-associated genes, as proved in RNA-seq data. According to the integrated analysis of MeRIP-seq and RNA-seq data, this study identified 16 significant DEGs with differential m^6^A peaks (*AMDHD1, CPM, DCTPP1, GIMAP1, GVIN1, HERC6, LOC100522040, LOC106510546, NFAT5, PIM3, PPARGC1B, RASSF2, SAPCD2, URB2, XRRA1,* and *ZC3HAV1L*), most of which are related to immune, metabolism, and virus infection. For example, porcine HERC6 catalyzes ISGylation of ISG15 for immune signaling to control viral replication and pathogenesis (Ba et al., 2024; Chu et al., 2024). GVIN1 protein is an interferon-induced large GTPases with antiviral activities (Klamp et al., 2003). Pim kinase3 (PIM3) is required at an early entry step of the HCV life cycle (Park et al., 2015). AMDHD1 has been shown to be involved in the histidine catabolic pathway, which is implicated in virus pathogenesis (Brosnan and Brosnan, 2020). AMDHD1 can inhibit the ubiquitination and degradation of the SMAD4 protein through binding to the MH2 domain to activate the TGF-β signaling pathway, which plays critical roles in maintaining intestinal mucosa homeostasis (Ma et al., 2024; Wang et al., 2023). In addition, the genetic variant of AMDHD1 is associated with vitamin D, which influences PEDV infection (Freitas et al., 2021; Yang et al., 2022). GIMAP1, a member of the GTPase of the immunity-associated protein family, is involved in the survival of naïve and activated immune cells (Webb et al., 2016; Datta et al., 2017). Recent studies found that nuclear-encoded mitochondrial genes involved in amino acid, lipid, and carbohydrate metabolism and ATP synthase show differential expression patterns across all tissues to regulate mitochondrial biogenesis for functional and energetic demand in large farm animals (Sadeesh and Lahamge, 2025; Sadeesh et al., 2025a; Sadeesh et al., 2025b). In this study, as a stimulator of transcription factors and nuclear receptor activities driving innate immune responses, mitochondrial biogenesis, and oxidative metabolism (Abe et al., 2024; Guak et al., 2022). Therefore, this study speculates that m^6^A methylation of PPARGC1B might contribute to its upregulation and could play a role in maintaining mitochondrial metabolism and restraining inflammation during PEDV infection. However, the precise mechanism, including which m^6^A reader proteins are involved and whether mRNA stability is affected, requires further experimental validation.

Another transcription factor, the nuclear factor of activated T cells 5 (NFAT5), is known to be involved in viral infections, including HIV-1, HBV, HCV, CVB3, LCMV, and VSV (Zhao et al., 2021). NFAT5 may activate NFκB/iNOS signaling, thereby inhibiting CVB3 replication and cardiomyocyte injury (Qiu et al., 2017). Moreover, NFAT5 was identified as the transcriptional suppressor of virus-induced type I interferons (IFN-I) production, blocking its excessive expression through binding the Ifnb1 enhancer and suppressing Ifnb promoter activity (Huerga Encabo et al., 2020). Interestingly, recent research showed that NFAT5 is hypomethylated in response to oxidative stress, and the m^6^A reader protein IGF2BP1 can interact with NFAT5 to regulate NFAT5 stabilization (Xu et al., 2023; Gao et al., 2023). HERC6 is a major E3 ligase for ISGylation, and a recent study showed that m^6^A regulators, such as IGF2BP2, mediated the m^6^A modification and upregulation of ISG15, leading to ISGylation of target genes (Ba et al., 2024; Han et al., 2026). In the present study, differential m^6^A methylation of HERC6 in PEDV-infected cells was observed. It is tempting to speculate that m^6^A methylation of HERC6 might influence its role in ISGylation during viral infection. However, this hypothesis requires direct experimental validation. Future studies, including gene knockdown or overexpression experiments and MeRIP-qPCR, are needed to determine whether m^6^A modification of HERC6 or other genes is functionally associated with PEDV infection and how m^6^A readers regulate the expression of differentially methylated genes.

Several limitations of this study should be acknowledged. First, all experiments were performed using LLC-PK1 cells, a porcine kidney epithelial cell line, rather than intestinal epithelial cells, which are the natural targets of PEDV infection *in vivo*. While LLC-PK1 cells are permissive to PEDV and widely used in PEDV research, the m^6^A methylation landscape and host responses may differ between kidney and intestinal epithelial cells. Future studies using porcine intestinal epithelial models, such as IPEC-J2 cells or primary intestinal epithelial cells, are needed to validate these findings. Second, this study is primarily descriptive and correlative; functional experiments (e.g., gene knockdown/overexpression and MeRIP-qPCR) are required to establish causal relationships between m^6^A methylation and the identified candidate genes. Despite these limitations, this study provides a valuable resource for understanding the potential role of m^6^A methylation in PEDV infection.

In summary, our results have characterized the dynamic landscape of host m^6^A methylation and confirmed the functional enrichment of m^6^A-methylated genes in immune-related pathways during PEDV infection, providing a theoretical basis for further research on m^6^A methylation in antiviral resistance. Moreover, combining m^6^A modification analysis and gene expression analysis, this study identified 16 differentially methylated and expressed genes (*AMDHD1, CPM, DCTPP1, GIMAP1, GVIN1, HERC6, LOC100522040, LOC106510546, NFAT5, PIM3, PPARGC1B, RASSF2, SAPCD2, URB2, XRRA1,* and *ZC3HAV1L*) associated with immune responses and metabolic regulation. This research enhances the understanding of the regulatory role of m^6^A methylation in PEDV infection, and these differentially methylated genes warrant further investigation as potential targets for antiviral therapy.

## Data Availability

The datasets presented in this study can be found in online repositories. The names of the repository/repositories and accession number(s) can be found at: https://ngdc.cncb.ac.cn/omix/release/OMIX014191, OMIX014191.
