# Supplementary material for: Landscape of N6-methyladenosine (m6A) methylation in porcine cells identifies candidate genes associated with porcine epidemic diarrhea virus infection
Source: Front Microbiol. 2026 May 18;17:1828985. doi: 10.3389/fmicb.2026.1828985 (PMC13222974; doi:10.3389/fmicb.2026.1828985)
Supplement: Supplementary file 1 [file Table_1.docx]

Supplementary Table S1 Gene primers for qRT-PCR

| Name | Primer sequence (5′ to 3′) |
| --- | --- |
| ZC3HAV1L | F: AACCGGGACTGCTGGTCTA |
|  | R: AGAGGGCTTCCCCTTTGTTG |
| DCTPP1 | F: CTCGCAGAACTCTTTCAGTGGA |
|  | R: GCCGGTTGATGTCCATTTTGG |
| URB2 | F: TCTGAAGCCGCTGGAGTATG |
|  | R: GTCATCTGCGCTTCCTTTGTC |
| HERC6 | F: TCCCTGGCATTATCAGAAGATGG |
|  | R: ATTATTCCCGCTGAGGGCCA |
| NFAT5 | F: GTAGTTGCTGCTGATGCTTCT |
|  | R: TTCGGGGTTGATGGATGCTG |
| PIM3 | F: CACCGACTTCGACGGCAC |
|  | R: CCCGCACACCATGTCATAGA |
| PPARGC1B | F: GGTGTACATTCGCAATCTCGC |
|  | R: GCACTTCTCGCCTCTCGTG |
| AMDHD1 | F: CCGCCTACATGCTGAGACTG |
|  | R: CCGTGAGTGTGAGACCTTCC |
| XRRA1 | F: GCATGTTCAAACACCCGCTC |
|  | R: TGCAGAACAGCTCCTAGTGG |
